# Deeply subwavelength giant monopole elastodynamic metacluster resonators

**DOI:** 10.1098/rspa.2022.0026

**Published:** 2022-07

**Authors:** Philip A. Cotterill, David Nigro, William J. Parnell

**Affiliations:** ^1^ Department of Mathematics, University of Manchester, Oxford Road, Manchester M13 9PL, UK; ^2^ Thales UK, 350 Longwater Avenue, Reading, Berkshire RG2 6GF, UK

**Keywords:** elastodynamic metamaterials, giant monopole resonance, metaclusters

## Abstract

The giant monopole resonance is a well-known phenomenon, employed to tune the dynamic response of composite materials comprising voids in an elastic matrix which has a bulk modulus much greater than its shear modulus, e.g. elastomers. This low frequency resonance (e.g. $\lambda_{p}/a\approx 100$ for standard elastomers, where $\lambda_{p}$ and a are the compressional wavelength and void radius, respectively) has motivated acoustic material design over many decades, exploiting the subwavelength regime. Despite this widespread use, the manner by which the resonance arising from voids in close proximity is affected by their interaction is not understood. Here, we illustrate that for planar elastodynamics (circular cylindrical voids), coupling due to near-field shear significantly modifies the monopole (compressional) resonant response. We show that by modifying the number and configuration of voids in a metacluster, the directionality, scattering amplitude and resonant frequency can be tailored and tuned. Perhaps most notably, metaclusters deliver a lower frequency resonance than a single void. For example, two touching voids deliver a reduction in resonant frequency of almost 16% compared with a single void of the same volume. Combined with other resonators, such metaclusters can be used as meta-atoms in the design of elastic materials with exotic dynamic material properties.

## 1. Introduction

Manipulating acoustic and elastic waves is of fundamental importance in numerous applications over a vast range of scales in science and engineering including sound control [1–3], energy harvesting [4,5], vibration isolation [6,7], subwavelength imaging [8,9], lensing [10], cloaking [11,12] and seismic applications [13]. Over the last two decades, a huge body of work has tackled a broad range of problems in this area via the design of acoustic [14] and elastodynamic *metamaterials* [15]. These are materials that have properties far beyond the naturally exhibited properties of commonly available media. Metamaterials rely on harnessing the interaction of a propagating wave with microstructural features or length scales. They typically rely on resonance so that the response is usually deeply subwavelength [16,17]. Indeed, this is the regime in which they are of most interest since it is in this domain that waves are hardest to control and manipulate.

The area of elastodynamics has traditionally received less attention than acoustics because the problems that arise there are typically vectorial, with shear waves inherently coupled to compressional waves. So whereas in acoustics the manipulation of the wave via resonance typically requires an understanding of the monopole and dipole resonance of an inclusion inside an inhomogeneous medium that has potential to act as a metamaterial, in elastodynamics, one needs to understand the monopole, dipole *and* quadrupole resonance to manipulate the full elastodynamic fields and the manner by which they interact. The original pioneering work of Liu *et al.* [16] involved placing a rigid inclusion in a softer shell, and then placing this inside another matrix, leading to a dipole resonance. Work since then has sought to tune this, and related resonances in order to appropriately control elastic waves [7,18,19].

A resonance that is often overlooked, but one that is potentially very useful in elastodynamic metamaterial applications, is the *giant monopole resonance* (GMR) [20–22]. This occurs for voids in elastic materials with Poisson ratio close to 1/2. A significant body of work associated with such resonances when voids are arranged in the configuration of a line array has been carried out by Ivansson [23,24] and more recently in [25], with a typical aim of absorbing incident elastic energy. The influence of more complex void configurations has, however, not been investigated and in particular the influence of geometric non-locality, i.e. when voids interact strongly due to multiple scattering. The statics case of closely interacting voids was considered some time ago now [26] but for the dynamic case it is not currently understood how the GMR is affected when two voids are in close proximity. It appears that there is potential therefore to modify and tune the GMR, enabling improved control over the elastodynamic field in the longer term, and providing the potential for a richer set of frequency-dependent dynamic material properties.

In [27], the concept of a *metacluster* was introduced. This is a collection of scatterers that is employed to tune the far-field response due to some incident field. Here, we therefore adopt this terminology and describe the resonators that we study as *giant monopole metacluster resonators*. Our principal objective is to illustrate that the configuration of circular cylindrical voids can have a significant effect on the resonant frequency and the associated planar far-field elastodynamic response. In §2, we introduce notation via a description of the elastic scattering formulation and the GMR for a single void prior to an asymptotic analysis of the low frequency regime, deriving a new approximation to the GMR frequency for a circular cylindrical void. This is important, because although an explicit expression is available for the resonance of a *spherical* void [22], such an expression cannot be written down for a cylindrical void. We then describe the multiple scattering formulation for an arbitrary configuration of J voids. In §3, we focus on the case of two interacting voids, the so-called *co-void metacluster*. In particular, we illustrate the impact of near-field coupling on the GMR, the far-field scattering pattern and deduce the accuracy of monopole approximations to the full field. Specific interest focuses on the fact that near-field interaction between voids can lead to a lower frequency resonance compared to the same volume of a single isolated void. We go on to introduce the concept of an *equivalent void*, which may be useful when incorporating such co-void resonators in multiple scattering formulations for metamaterials. In §4, we discuss more complex configurations of void metaclusters, including the tri- and quad-void metaclusters. We close in §5 with conclusions.

## 2. Elastodynamic scattering and the giant monopole resonance

We consider linear elastic wave propagation in two dimensions (the *x*–*y* plane) of an unbounded, isotropic medium. Navier’s equations therefore govern the elastic displacements. We assume that displacements exhibit time-harmonic dependence, with angular frequency ω, of the form $ℜ(u(x)\;e^{-i\omega t})$. Here, x is the position vector and t denotes time, and we thus work with the complex valued displacement u(x). It is convenient to introduce the Helmholtz potentials φ and ψ in the form

2.1 $$\text{u}=\frac{1}{k_{s}^{2}}\nabla \times (\psi \hat{\text{z}})-\frac{1}{k_{p}^{2}}\nabla \varphi ,$$

where we have introduced the compressional and shear wavenumbers $k_{p}$ and $k_{s}$ via the standard relations $k_{p}^{2}=\omega^{2}\rho /(\lambda +2\mu )$ and $k_{s}^{2}=\omega^{2}\rho /\mu$, λ and μ are the usual Lamé constants, with μ being the shear modulus and ρ is the mass density. The unit vector in the z-direction (out of the *x*–*y* plane of displacement polarization) is denoted by $\hat{z}$. This framework allows us to work with the compressional and shear scalar potentials φ and ψ, respectively, both of which satisfy Helmholtz’s equation

2.2 $$(\nabla^{2}+k_{p}^{2})\varphi =0\;\mathrm{and}\;(\nabla^{2}+k_{s}^{2})\psi =0.$$

### (a) Scattering from a single circular inclusion

The focus will be on scattering from circular inclusions of radius a and it is therefore convenient to introduce polar coordinates related to x in the usual manner x=rcos⁡θ,y=rsin⁡θ and for a single scatterer, we locate the origin of the coordinate system at the centre of the scatterer. Prescribed incident fields comprising both compressional and shear sources are conveniently expressed as

2.3 $$\varphi^{\text{in}}=\sum_{m=-\infty }^{\infty }A_{m}J_{m}(k_{p}r)\;e^{\text{i}m\theta }\;\mathrm{and}\;\psi^{\text{in}}=\sum_{m=-\infty }^{\infty }C_{m}J_{m}(k_{s}r)\;e^{\text{i}m\theta },$$

while the scattered fields, which satisfy the radiation condition as r→∞, are written in r>a as

2.4 $$\varphi^{\text{sc}}=\sum_{m=-\infty }^{\infty }B_{m}H_{m}^{\left(1\right)}(k_{p}r)\;e^{\text{i}m\theta }\;\mathrm{and}\;\psi^{\text{sc}}=\sum_{m=-\infty }^{\infty }D_{m}H_{m}^{\left(1\right)}(k_{s}r)\;e^{\text{i}m\theta },$$

where $J_{m}(z)$ denotes the Bessel function of order m and of the first kind and $H_{m}^{\left(1\right)}(z)$ is the Hankel function of the first kind, of order m. Total fields in the domain r>a are therefore represented via $\varphi =\varphi^{\text{in}}+\varphi^{\text{sc}}$ and $\psi =\psi^{\text{in}}+\psi^{\text{sc}}$.

Boundary conditions on r=a enable the relationship between the scattered and (known) incident coefficients to be determined via what has become known as the *T-matrix* [28]

2.5 $$\left[\begin{array}{c} B_{m}\\ D_{m} \end{array}\right]={\text{T}}^{\left(m\right)}\left[\begin{array}{c} A_{m}\\ C_{m} \end{array}\right].$$

The T-matrix $T^{\left(m\right)}$ for any scattering order m is thus a 2×2 matrix, encompassing the scatterer’s mechanical properties and it is independent of the incident field. Results determined in terms of the T-matrix are therefore general and in particular as we shall see in §2d, the T-matrix is efficiently employed in multiple scattering problems.

Here, we are interested in scattering from *voids*, and thus

2.6 $$\sigma_{rr}=\sigma_{r\theta }=0,\;\text{on }r=a,$$

where $\sigma_{rr}$ and $\sigma_{r\theta }$ are the longitudinal-radial and shear components of the Cauchy stress with respect to a polar coordinate basis. They can be expressed in terms of the scalar potentials as follows,

2.7 $$\frac{\sigma_{rr}}{\lambda +2\mu }=\varphi +2\xi \left[\frac{1}{k_{s}^{2}}\frac{\partial }{\partial r}\left(\frac{1}{r}\frac{\partial \psi }{\partial \theta }\right)+\frac{1}{k_{p}^{2}}\left(\frac{1}{r}\frac{\partial \varphi }{\partial r}+\frac{1}{r^{2}}\frac{\partial^{2}\varphi }{\partial \theta^{2}}\right)\right]$$

and

2.8 $$\frac{\sigma_{r\theta }}{\lambda +2\mu }=\xi \left\{\psi +2\left[\frac{1}{k_{s}^{2}}\left(\frac{1}{r}\frac{\partial \psi }{\partial r}+\frac{1}{r^{2}}\frac{\partial^{2}\psi }{\partial \theta^{2}}\right)-\frac{1}{k_{p}^{2}}\frac{\partial }{\partial r}\left(\frac{1}{r}\frac{\partial \varphi }{\partial \theta }\right)\right]\right\},$$

where λ+2μ has been factored out to emphasize their relative scaling via the rigidity parameter $\xi =\mu /(\lambda +2\mu )=k_{p}^{2}/k_{s}^{2}$, which is much less than unity for materials of interest. Further, we note that the polar displacement components are given by

2.9 $$u_{r}=\frac{1}{k_{s}^{2}r}\frac{\partial \psi }{\partial \theta }-\frac{1}{k_{p}^{2}}\frac{\partial \varphi }{\partial r}\;\mathrm{and}\;u_{\theta }=-\frac{1}{k_{s}^{2}}\frac{\partial \psi }{\partial r}-\frac{1}{k_{p}^{2}r}\frac{\partial \varphi }{\partial \theta }.$$

Imposing the boundary conditions (2.6) upon the above expressions allows the T-matrix components associated with a single circular void to be determined; they are stated in appendix A.

We are interested in the case when the incident field is purely compressional, i.e. we take $C_{m}=0$ for all m and furthermore we consider the incident field to be a plane wave of the form

2.10 $$\begin{array}{rl} \varphi^{\text{in}}(\text{x}) & \;=A\;e^{\text{i}k_{p}(x\cos\theta_{p}+y\sin\theta_{p})} \end{array}$$

**Figure 1.**
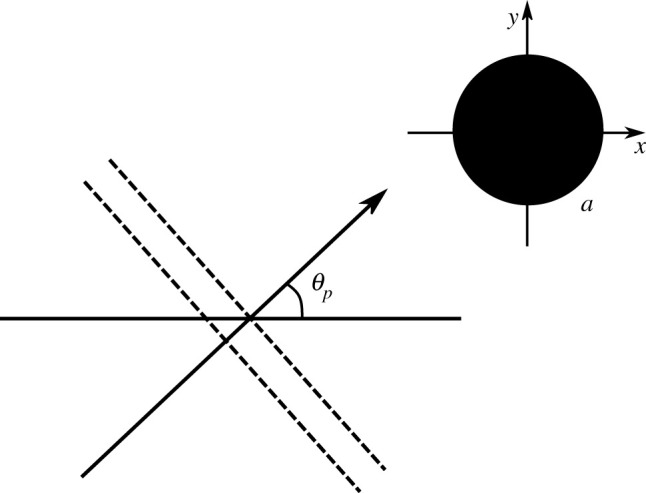
The giant monopole resonance is associated with scattering from a void inside an elastic medium where λ≫μ, such as an elastomer.

2.11 $$\begin{array}{rl} & \;=A\sum_{m=-\infty }^{\infty }{\text{i}}^{m}J_{m}(k_{p}r)\;e^{\text{i}m(\theta -\theta_{p})}, \end{array}$$

where $\theta_{p}$ is the angle subtended from the positive x-axis, see figure 1.

In (2.3), we thus make the connection

2.12 $$A_{m}=A{\text{i}}^{m}\;e^{-\text{i}m\theta_{p}}\;\mathrm{and}\;C_{m}=0.$$

### (b) The giant monopole resonance

It is well known that when insonified by a compressional wave, a void within an elastomeric medium (λ≫μ, i.e. ξ≪1) exhibits a strong resonance in its surface radial displacement component at angular order zero (m=0). This GMR [20–22] occurs at $k_{s}a=O(1)$ but given that $k_{p}a=k_{s}a\sqrt{\xi }$ and ξ≪1, this amounts to having $k_{p}a\ll 1$ and so is deeply subwavelength from the point of view of the compressional wave. This resonance therefore has significant potential in elastodynamic metamaterial applications.

Let us stress that throughout the paper, we frequently plot expressions as functions of $k_{s}a$, due to the fact that such expressions scale better in this parameter. Where possible however we indicate the associated $\lambda_{p}/a$ value at resonance, indicating its deeply subwavelength nature.

**Figure 2.**
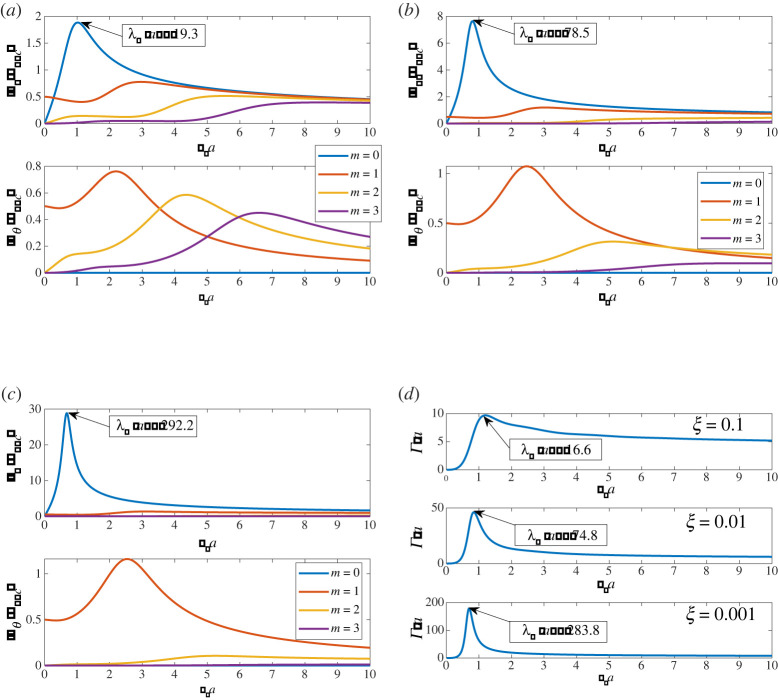
An illustration of the giant monopole resonance (GMR) associated with an isolated circular void in a medium with rigidity parameter ξ. In (*a*–*c*), the magnitudes of the Fourier decomposed void-surface-displacements $\left(u_{r},u_{\theta }\right)$ are plotted for the four lowest angular orders (m=0→3) as a function of $k_{s}a$ at each of three values of ξ. In these plots, the GMR is seen as a peak in the monopole (m=0) term of radial displacement whereas in (*d*) the resonance is defined by the peak in the scattering cross-section Γ. (*a*) ξ=0.1, (*b*) ξ=0.01, (*c*) ξ=0.001 and (*d*) scattering cross-section. (Online version in colour.)

There are at least three ways that the GMR resonance can be defined; the first is via the magnitude of displacements on the surface of the void. This is illustrated in figure 2*a*–*c*, in which we plot the four lowest orders (m=0,1,2,3) of the surface radial- (upper window) and circumferential- (lower window) displacements as a function of $k_{s}a$ when the void is insonified by a plane compression wave. The plotted surface displacements are normalized on those of the incident field, and are shown for three different values of rigidity: ξ=0.1,0.01 and 0.001, noting that $u_{\theta }=0$ for m=0 because the scattering of compression and shear waves is decoupled. The GMR is evident in the m=0 curve of figure 2*a* at $k_{s}a\approx 1$; it becomes more pronounced and moves to lower frequencies as ξ reduces, i.e. as the substrate becomes softer. In all three figures, $u_{r}^{\left(m\right)}$ and $u_{\theta }^{\left(m\right)}$ are almost identical at very small values of $k_{s}a$ (except for angular order zero) but as $k_{s}a$ approaches and then passes through the GMR value, their behaviours begin to deviate.

It is notable that as the material becomes softer, the low frequency behaviour of $u_{r}$ is increasingly dominated by the monopole (m=0) and dipole (m=1) modes, with this dominance being extremely pronounced for the soft (ξ=0.001) material shown in figure 2*c*. As $k_{s}a$ increases, the contribution from higher order modes becomes more significant and the effect is stronger for more rigid materials. This effect arises for softer materials also, but at higher values of $k_{s}a$ than are shown in the plots. Similar behaviour is observed for $u_{\theta }$, except that the magnitude at fixed rigidity is much smaller than the radial displacement and furthermore the monopole mode is absent as noted above.

A second way to define the resonance is via the dependence of the T-matrix on $k_{p}a$. We provide details of this below. A third approach to defining the resonance is via the *scattering cross-section* (SCS), Γ [29]. For completeness, we provide details of this in appendix B and in particular, we specify two formulae for its calculation. These expressions are equivalent for a non-absorbing scatterer. The SCS is a global measure of scattering, incorporating all angular orders, but for soft materials this measure is dominated by the monopole contribution as can be seen in figure 2*d* where the SCS, scaled on the void radius a is plotted as a function of $k_{s}a$ for the three values of ξ considered above.

The maximum of the SCS is particularly striking for the softest material considered, i.e. ξ=0.001, illustrated in the lowest plot in figure 2*d*.

The distinction between defining the resonance via the magnitude of surface displacements or via the SCS is twofold. Firstly, the former is a measure of the *total* displacement, whereas the latter is a measure of scattered energy alone. Furthermore, as we shall show, the SCS can be easily generalized to multiple voids and other configurations of scatterers (e.g. arrays) and therefore it is this measure that we shall employ throughout the paper to understand the importance of the GMR for collections of interacting voids.

That the peaks in SCS in figure 2*d* are mainly due to the monopole resonance can be seen from the form of (B 18) with J=1, noting that for an isolated, circular scatter $B_{m}^{\left(1\right)}/A_{m}^{\left(1\right)}\equiv T_{11}^{\left(m\right)}$, whence,

2.13 $$\Gamma =-\frac{4}{k_{p}}\sum_{m=-\infty }^{\infty }ℜ\{T_{11}^{\left(m\right)}\}.$$

**Figure 3.**
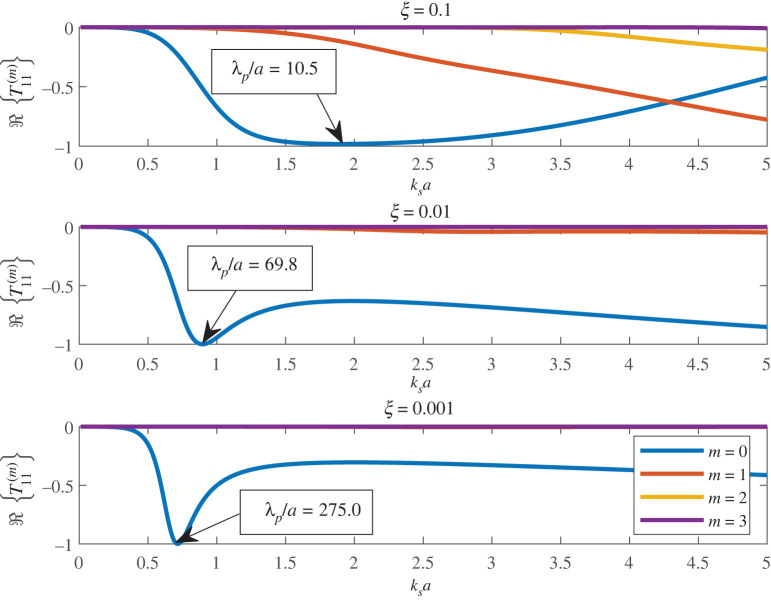
$ℜ\{T_{11}^{\left(m\right)}\}$ for various angular orders, as indicated in the legend. The value of rigidity is indicated for each sub-plot along with the value of $\lambda_{p}/a$ at the first minimum value of $ℜ\{T_{11}^{\left(0\right)}\}$; we note these values are somewhat smaller than the GMR as defined by the SCS in figure 2. (Online version in colour.)

The behaviour of $ℜ\{T_{11}^{\left(m\right)}\}$ is illustrated in figure 3, using the formulae given in appendix A. When $k_{s}a=O(1)$, $T_{11}^{\left(0\right)}$ largely dominates the other angular orders for the rigidity parameters shown. Thus,

2.14 $$\Gamma \approx -\frac{4}{k_{p}}ℜ\{T_{11}^{\left(0\right)}\},\;\text{for }k_{s}a=O(1),$$

and, for a given value of ξ, the low frequency peak of $-T_{11}^{\left(0\right)}$ effectively defines the GMR frequency. Note, however, that the peak in the SCS generally occurs at a lower frequency due to the factor of $k_{p}$ in the denominator of (2.13) and (2.14), hence the distinction between the GMR as defined by the T-matrix and SCS.

From (A 1), we find that

2.15 $$T_{11}^{\left(0\right)}=-\frac{J_{0}(k_{p}a)-(2\xi /k_{p}a)J_{1}(k_{p}a)}{H_{0}^{\left(1\right)}(k_{p}a)-(2\xi /k_{p}a)H_{1}^{\left(1\right)}(k_{p}a)}.$$

Given the form of (2.15), it is evident that $|T_{11}^{\left(0\right)}|$ has a maximum value of 1, which occurs whenever

2.16 $$Y_{0}(k_{p}a)-\frac{2\xi }{k_{p}a}Y_{1}(k_{p}a)=0.$$

Now, (2.16) poses an infinite number of zeros that become periodically spaced as $k_{p}a$ becomes large, occurring at $k_{p}a=\kappa_{n}∼n\pi +\pi /4$ for large integer values of n. At these zeros $T_{11}^{\left(0\right)}=-1$. For ξ≲0.075, (2.16) possesses a low frequency zero in the vicinity of $k_{s}a∼1$ and this gives rise to the GMR. We shall discuss this asymptotic regime in the next section. Denoting the value of $k_{p}a$ associated with this zero by $\kappa_{0}$, and setting $T_{11}^{\left(0\right)}=-1$, we see from (2.14) that $\Gamma /a\approx 4/\kappa_{0}$ at the GMR. For ξ≳0.075, the low frequency zero of (2.16) vanishes and instead becomes a minimum. It is evident from the upper plot of figure 2*d* that even so, it may still give rise to a substantial low frequency peak in the cross-section.

### (c) Asymptotic approximations for the resonance

In this section, we use the fact that $k_{p}a\ll 1$ to seek an approximate solution to (2.16). We start by noting that (2.16) can be rewritten in the form

2.17 $$(1-\xi )Y_{0}(k_{p}a)-\xi Y_{2}(k_{p}a)=0.$$

Before making progress on the asymptotic solution, we can analyse the exact equation further. It is worth noting that for small enough values of ξ and at low enough frequency (i.e. $k_{p}a⪅1$), there are two resonances. As ξ increases, the lower resonance frequency increases whereas the upper resonance frequency decreases. They eventually merge at a specific value of ξ, say $\xi_{m}$, above which these two resonances no longer exist. From (2.17), we can express ξ as a function of $k_{p}a$ at resonance,

2.18 $$\xi =\frac{Y_{0}(k_{p}a)}{Y_{0}(k_{p}a)+Y_{2}(k_{p}a)}.$$

Differentiating with respect to $k_{p}a$ gives

2.19 $$\frac{\text{d}\xi }{\text{d}k_{p}a}=\frac{Y_{0}^{\prime }(k_{p}a)Y_{2}(k_{p}a)-Y_{0}(k_{p}a)Y_{2}^{\prime }(k_{p}a)}{{\left(Y_{0}(k_{p}a)+Y_{2}(k_{p}a)\right)}^{2}}.$$

At the maximum, the numerator is equal to zero which gives

2.20 $$Y_{0}^{\prime }(k_{p}a)Y_{2}(k_{p}a)-Y_{0}(k_{p}a)Y_{2}^{\prime }(k_{p}a)=0.$$

The solution to this equation can be found numerically and is approximately $k_{p}a\approx 0.53$ which gives the maximum value of ξ above which the GMR does not exist as $\xi_{m}\approx 0.076$. We can summarize the behaviour as follows. For $0\leq \xi \leq \xi_{m}$, there are two resonances and therefore two branches. As $\xi \to 0^{+}$, the lower branch tends to 0 whereas the upper branch tends to the first zero of the Bessel function of the second kind of order zero, i.e. $y_{\left(0,1\right)}\approx 0.894$. As ξ increases, the upper resonance decreases whereas the lower resonance increases before merging at $\xi_{m}\approx 0.076$ and $k_{p}a\approx 0.53$.

We now focus on finding an approximate solution for the resonance frequency. Assuming that $k_{p}a\ll 1$, the small argument asymptotic expansion for $Y_{0}$ and $Y_{2}$ gives

2.21 $$2(1-\xi )\log(k_{p}a)+\xi {\left(\frac{2}{k_{p}a}\right)}^{2}+\xi +2(\gamma -\log(2))(1-\xi )+O({\left(k_{p}a\right)}^{2})=0,$$

where γ is Euler’s constant. This equation generally needs to be solved numerically, although some progress can be made analytically. First introduce $z=4\xi /((1-\xi ){\left(k_{p}a\right)}^{2})$ so that the previous equation can be recast in the form

2.22 $$\log\left(\frac{1}{z}\right)+z=-\left(2\gamma +\frac{\xi }{1-\xi }+\log\left(\frac{\xi }{1-\xi }\right)\right)=-C(\xi ).$$

For the rest of the analysis, we will drop the ξ dependency of C to simplify the notation. The solution can be written in terms of the Lambert W function [30] with argument −exp⁡(C), which is real and negative. For real negative arguments larger than −1/e, the Lambert W function has two real negative branches denoted $W_{-1}$ and $W_{0}$. Hence we have two solutions, $z_{0}^{+}=W_{0}(-\exp\left(C\right))$ and $z_{0}^{-}=W_{-1}(-\exp\left(C\right))$ as long as −exp⁡(C)≥−1/e. Therefore, the solution in terms of wavenumbers has an upper (+) and lower (−) branch, specified by

2.23 $$k_{p}a^{\left(+\right)}={\left(\frac{4\xi }{-(1-\xi )W_{0}(-\exp(C))}\right)}^{1/2}$$

and

2.24 $$k_{p}a^{\left(-\right)}={\left(\frac{4\xi }{-(1-\xi )W_{-1}(-\exp(C))}\right)}^{1/2}.$$

**Figure 4.**
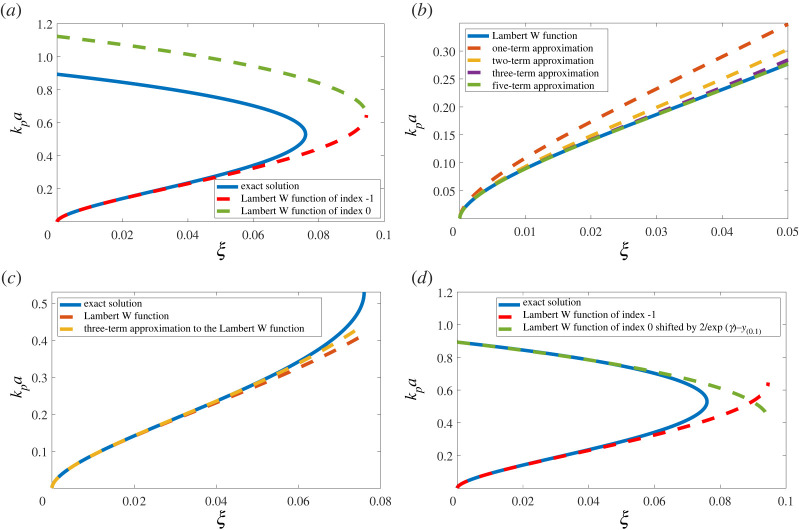
Comparison between the exact solution to (2.17) and various approximations, i.e. (*a*) the expressions from (2.24) and (2.23); (*b*) (2.24) with various truncations of equation (2.26); (*c*) the three-term approximation using equation (2.25); (*d*) the expressions (2.24) and (2.23) but with $k_{p}a^{\left(+\right)}$ shifted down by $\left(2/\exp(\gamma )-y_{\left(0,1\right)}\right)$. (Online version in colour.)

In the asymptotic regime, the resonance only exists for −exp⁡(C)≥−1/e which is equivalent to C≤−1. In terms of ξ, this corresponds to ξ⪅0.0945 and we note that this value is larger than that found using the exact expression. This is mostly due to the fact that, for such large values of ξ, the wavenumber $k_{p}a$ is no longer small and therefore the asymptotic solution is not valid. A comparison between the exact solution to equation (2.17) and the expressions from (2.24) and (2.23) can be found in figure 4*a*. The agreement on the lower branch below ξ≈0.05 and $k_{p}a\approx 0.3$ is excellent and illustrates the efficacy of asymptotic approximations in certain regimes.

As we are interested in the small wavenumber regime, we must pay particular attention to the lower branch of the solution. Furthermore, ξ is much smaller than unity. In this limit, the argument in the Lambert W function of index −1 becomes $0^{-}$ and therefore one can use the approximation ([31] eqn. 4.13.11)

2.25 $$\begin{array}{rl} W(-1,-\exp(C)) & \;=C-\log(-C)+\frac{\log(-C)}{C}+\frac{1}{2}{\left(\frac{\log(-C)}{C}\right)}^{2}-\frac{\log(-C)}{C^{2}}+O\left({\left(\frac{\log(-C)}{-C}\right)}^{3}\right). \end{array}$$

This yields the following approximation to the resonant frequency:

2.26 $$k_{p}a^{\left(-\right)}\approx {\left(\frac{4\xi }{(\xi -1)\left(C-\log(-C)+(\log(-C)/C)+\frac{1}{2}{\left(\frac{\log(-C)}{C}\right)}^{2}-(\log(-C)/C^{2})\right)}\right)}^{1/2}.$$

A comparison of the exact solution to the asymptotic equation (2.24) and various truncations of equation (2.26) including one, two, three and five terms can be found in figure 4*b*. As should be expected, as the number of terms increases, the accuracy of the various approximations improves, and with five terms the two curves are indistinguishable at the scale shown. However, when comparing the various approximations based on (2.26) with the exact solution to (2.17), we note that the three-term approximation matches the exact solution better than the five-term approximation and (2.24). This result is highlighted in figure 4*c*. Therefore, we suggest using the following approximation for the lower branch of the solution:

2.27 $$k_{p}a^{\left(-\right)}\approx {\left(\frac{4\xi }{\left(\xi -1)(C-\log(-C)+(\log(-C)/C)\right)}\right)}^{1/2}.$$

Finally, it is worth noting that although the asymptotic solutions (2.23) and (2.24) are not directly applicable over the entire range of wavenumbers, they still capture the existence and behaviour of the upper branch (figure 4*a*) of the exact solution. Furthermore, as $\xi \to 0^{+}$, the upper branch of the asymptotic solution increases to $k_{p}a=2/\exp(\gamma )$. It is an interesting observation that the upper branch of the asymptotic solution behaves in a similar fashion to the upper branch of the exact solution but shifted by $\left(2/\exp(\gamma )-y_{\left(0,1\right)}\right)$ as can be seen in figure 4*d*. Once the expression from (2.23) is shifted down by $\left(2/\exp(\gamma )-y_{\left(0,1\right)}\right)$, the two curves overlap for ξ below 0.05.

Let us now consider the formulation for scattering from multiple voids.

### (d) Scattering from J voids

We have seen that the GMR associated with a single void is well defined provided ξ is sufficiently small. Furthermore, as ξ is reduced, the resonance becomes stronger and is more narrow band. For small ξ, the SCS near resonance is completely dominated by the monopole contribution of $T_{11}^{\left(0\right)}$ and clearly due to symmetry there is no dependence on angle of incidence.

Our interest here is in how the scattering resonance is affected by the presence of many scatterers in various configurations, generating coupling via multiple scattering between the voids. This represents an opportunity for the control of elastic waves via a specified configuration of voids. We term this a *void metacluster*, with terminology inspired by [27]. Furthermore, given that in general, metamaterials consist of multiple subwavelength scatterers, we consider that the above are important sub-problems from which to potentially fabricate novel elastodynamic metamaterials for the control of elastic waves.

To understand the complex coupling that can occur between voids, in order to modify the GMR response and to control elastic waves, here we summarize the formulation for scattering from J circular voids of arbitrary size, although in results presented later attention is restricted to identical voids. This allows us to write down an expression for the efficient calculation of the SCS from multiple voids.

Consider the case of J circular voids, with centres $x_{j}=(x_{j},y_{j})$ and radii $a_{j}$, for j=1,2,…,J, that are insonified by the plane compression wave defined in (2.10) to (2.12). The total potential functions may thus be written [32]

2.28 $$\varphi (\text{x})=\varphi^{\text{in}}(\text{x})+\sum_{j=1}^{J}\sum_{m=-\infty }^{\infty }B_{m}^{\left(j\right)}H_{m}^{\left(1\right)}(k_{p}r_{j})\;e^{\text{i}m\theta_{j}}$$

and

2.29 $$\psi (\text{x})=\sum_{j=1}^{J}\sum_{m=-\infty }^{\infty }D_{m}^{\left(j\right)}H_{m}^{\left(1\right)}(k_{s}r_{j})\;e^{\text{i}m\theta_{j}},$$

where $B_{m}^{\left(j\right)}$ and $D_{m}^{\left(j\right)}$ are respectively the scattering coefficients associated with the jth scatterer and mth mode. Local polar coordinates $\left(r_{j},\theta_{j}\right)$ are defined via $x-x_{j}=r_{j}(\cos\theta_{j},\sin\theta_{j})$. Within the vicinity of the ith scatterer say, we may write the total field in the form

2.30 $$\varphi =\sum_{m=-\infty }^{\infty }(A_{m}^{\left(i\right)}J_{m}(k_{p}r_{i})+B_{m}^{\left(i\right)}H_{m}^{\left(1\right)}(k_{p}r_{i}))\;e^{\text{i}m\theta_{i}}$$

and

2.31 $$\psi =\sum_{m=-\infty }^{\infty }(C_{m}^{\left(i\right)}J_{m}(k_{s}r_{i})+D_{m}^{\left(i\right)}H_{m}^{\left(1\right)}(k_{s}r_{i}))\;e^{\text{i}m\theta_{i}}$$

for some coefficients $A_{m}^{\left(i\right)}$ and $C_{m}^{\left(i\right)}$, which may be obtained by expanding (2.28) and (2.29) within the ith local coordinate system and employing Graf’s addition theorem for j≠i [32]. One can thus show that

2.32 $$A_{m}^{\left(i\right)}=A^{\left(i\right)}{\text{i}}^{m}\;e^{-\text{i}m\theta_{p}}+\sum_{n}\sum_{j\neq i}B_{n}^{\left(j\right)}\;e^{\text{i}(n-m)\phi_{i}^{\left(j\right)}}H_{n-m}^{\left(1\right)}(k_{p}R_{i}^{\left(j\right)})$$

and

2.33 $$C_{m}^{\left(i\right)}=\sum_{n}\sum_{j\neq i}D_{n}^{\left(j\right)}\;e^{\text{i}(n-m)\phi_{i}^{\left(j\right)}}H_{n-m}^{\left(1\right)}(k_{s}R_{i}^{\left(j\right)}),$$

where

**Figure 5.**
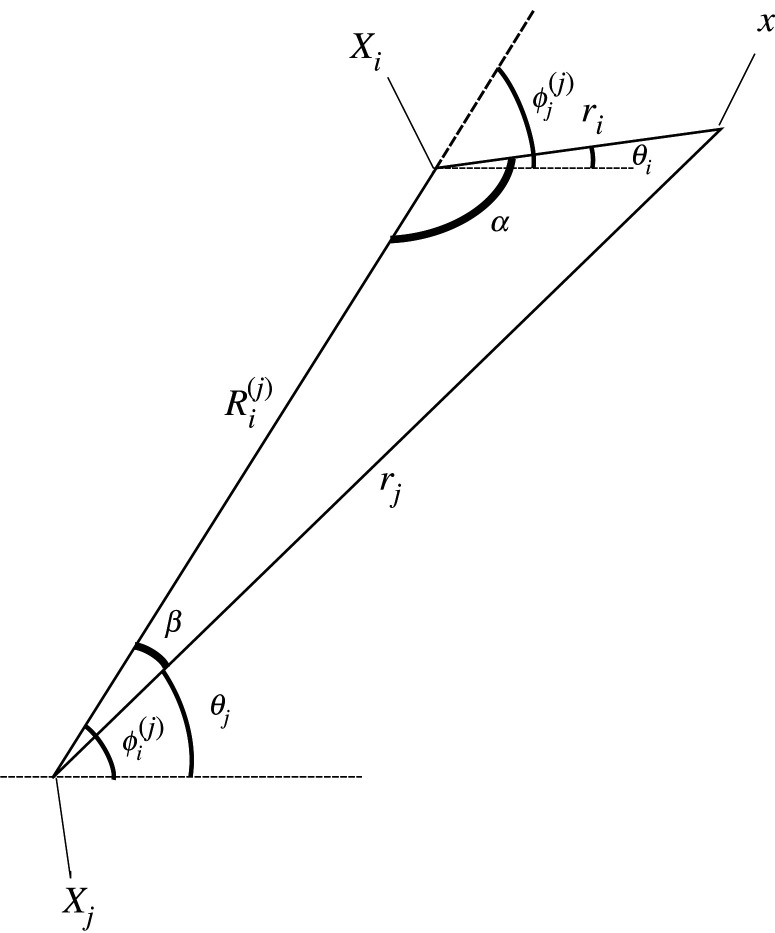
Relationship between the ith and jth local coordinate systems.

2.34 $$A^{\left(i\right)}=A\;e^{\text{i}k_{p}(x_{i}\cos\theta_{p}+y_{i}\sin\theta_{p})},$$

and $R_{i}^{\left(j\right)}$ and $\phi_{i}^{\left(j\right)}$ are the polar coordinates of the ith void expressed within the jth local coordinate system (figure 5).

The scattering coefficients for each void are related by the T-matrix of (2.5); for the ith scatterer we write this as

2.35 $$\left[\begin{array}{c} B_{m}^{\left(i\right)}\\ D_{m}^{\left(i\right)} \end{array}\right]={\text{T}}^{\left(m,i\right)}\left[\begin{array}{c} A_{m}^{\left(i\right)}\\ C_{m}^{\left(i\right)} \end{array}\right],$$

noting that for circular voids, the only difference between the T-matrix of each scatterer is associated with the void radius. Using this in conjunction with (2.32) and (2.33) allows the determination of the scattering coefficients for all voids, given their specific configuration.

In the next section, we consider various configurations of voids, beginning with the case of two voids separated by a distance d. We then go on to consider more complex void systems. The specific interest in each case is to understand how the cluster configuration affects the SCS, the resonant frequency, and far-field directivity pattern as a function of incident angle. For the co-void case, we consider in detail the significance of the spacing d/a. We also discuss how two alternative monopole approximations compare to the true elastic wave field.

## 3. The co-void metacluster

**Figure 6.**
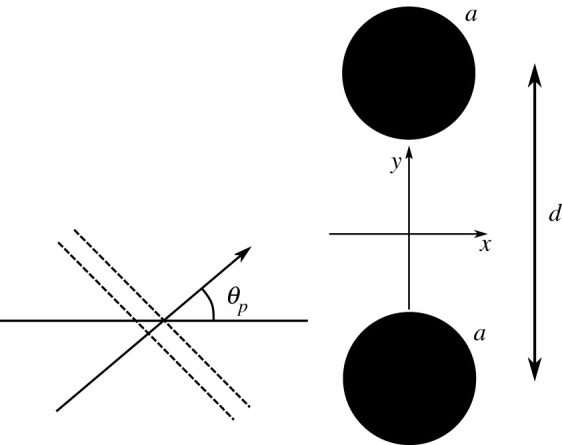
Illustrating compressional wave scattering from a co-void metacluster.

With reference to figure 6, we consider scattering of a plane compressional wave from two voids with the same radius a and whose centres are located at $\left(x_{1}=0,y_{1}=-d/2\right)$ and $\left(x_{2}=0,y_{2}=d/2\right)$. We use the term *co-void metacluster* for this configuration, and assess how the scaled void spacing d/a, and the incident angle $\theta_{p}$, affect scattering and the GMR, as defined by the peak SCS.

### (a) Impact of coupling on the giant monopole resonance

**Figure 7.**
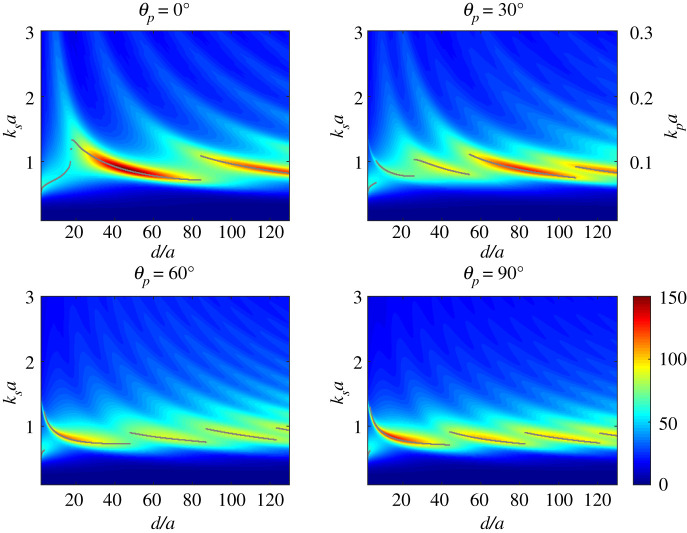
Non-dimensionalized elastic scattering cross-section, Γ/a, versus $k_{s}a$ and d/a for two voids insonified by a compressional wave, showing a range of incidence angles from $\theta_{p}=0^{\circ }$ (upper-left) to $\theta_{p}=90^{\circ }$ (bottom-right). In all plots ξ=0.01, and the grey markers indicate the values of $k_{s}a$ at which Γ/a peaks for each value of d/a. We have also added the $k_{p}a$ scale on the top right figure to illustrate that the resonance remains low frequency for the compressional wave. (Online version in colour.)

In figure 7, we plot the scaled SCS, Γ/a, calculated via (B 18) and (B 19) with J=2. We illustrate this as a heat map as a function of d/a and $k_{s}a$ for incident angles $\theta_{p}=0,30,60$ and $90^{\circ }$ and with ξ=0.01. We also indicate the $k_{s}a$ value at which the maximum value of the SCS occurs, given the value of d/a, interpreting this as the GMR for the co-void system.

At larger values of d/a, Γ/a peaks at roughly the same frequency as a single void, converging to $k_{s}a\approx 0.84$ ($\lambda_{p}/a\approx 74.8$) as d/a→∞ as should be expected. At smaller values of d/a however, and particularly as d/a→2, which corresponds to touching voids, the behaviour changes significantly. For all angles of incidence, when d/a→2, the peak resonance shifts to $k_{s}a\approx 0.5$ ($\lambda_{p}/a\approx 125.7$). A single circular-cylindrical scatterer having the same volume as the touching co-void metacluster would have a radius of $\sqrt{2}a$, and a resonant frequency value equivalent to $k_{s}a\approx 0.84/\sqrt{2}\approx 0.59$ ($\lambda_{p}/a\approx 106.5$). Thus the touching co-void metacluster gives a reduction in resonant frequency of almost 16% compared with a single void of the same volume. Conversely, the radius of a single void matching the resonant frequency of the touching co-void system would be $\tilde{a}\approx (0.84/0.5)a\approx 1.68a$. Hence the resonant frequency of a single void can be matched by two touching voids but with a volume reduction of almost 30%. This can therefore be considered as either a lowering of the resonance for the same volume or it allows the same resonance to be achieved with less void volume. We discuss this concept further in §3d, however, it should be stressed that the mechanism for inducing this effect is local shear wave scattering, which is weak in the far-field but strong locally.

Figure 7 illustrates that for smaller values of d/a, the dependence on incidence angle is rather complex. For d/a less than approximately 20, at normal incidence ($\theta_{p}=0$), the peak magnitude of the SCS reduces and bifurcates. The upper branch rises rapidly in frequency as d/a→2 and reduces in magnitude, whereas the lower branch converges to $k_{s}a\approx 0.5$ as d/a→2, and increases somewhat in magnitude, becoming the GMR over this parameter range (as defined by maximum SCS). Similar behaviour is seen at other angles of incidence but the upper branch becomes more dominant over larger ranges of d/a as we increase $\theta_{p}$. It also increases less in frequency as d/a→2.

### (b) Impact of coupling on far-field directivity

**Figure 8.**
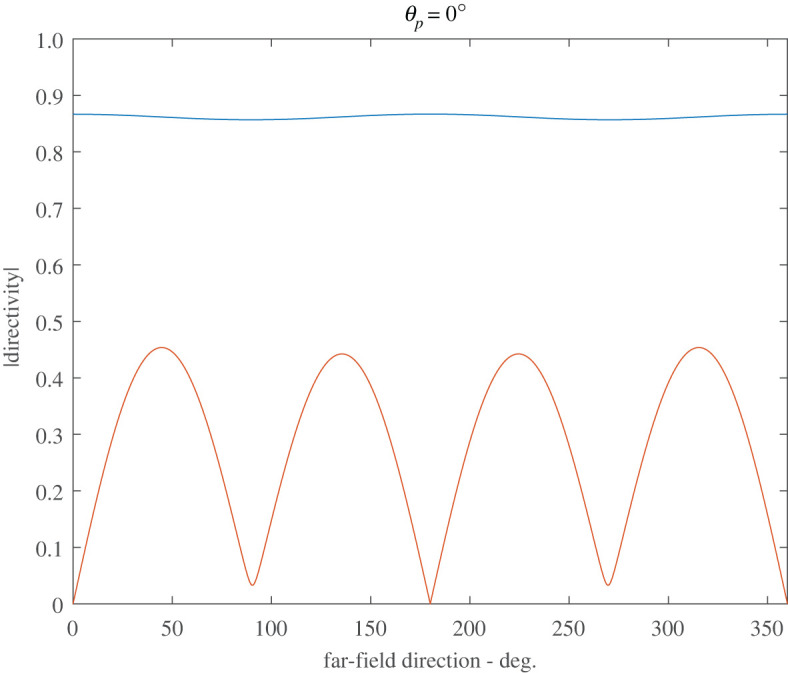
Far-field directivity patterns of σ/(λ+2μ) for two touching voids at $k_{s}a=0.5\;(\lambda_{p}/a=125.7)$, and ξ=0.01, for normal incidence $\theta_{p}=0^{\circ }$. The blue curve is associated with the dilational-stress, $\sigma_{D}=\text{tr}(\sigma )/3$, and the red curve is associated with the polar shear-stress, $\sigma_{r\theta }$. Both are scaled on λ+2μ. (Online version in colour.)

**Figure 9.**
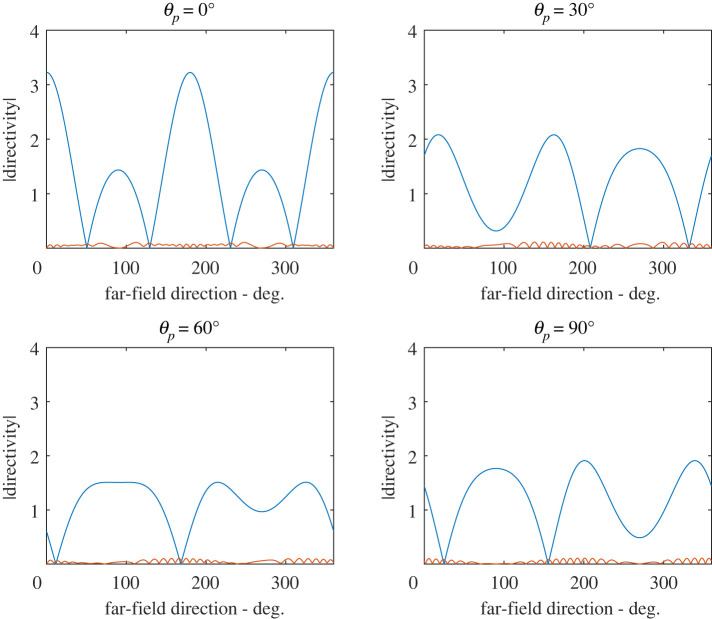
Far-field directivity patterns of σ/(λ+2μ) for two voids at $k_{s}a=0.86\;(\lambda_{p}/a=73.1),d/a=47.3$, the configuration where the co-void cross-section is a maximum at normal incidence. The blue curves are for dilational-stress, $\sigma_{D}=\text{tr}(\sigma )/3$, and the red curves are for polar shear-stress, $\sigma_{r\theta }$. Four angles of incidence are illustrated: from $\theta_{p}=0^{\circ }$ at top left to $\theta_{p}=90^{\circ }$ at bottom right. ξ=0.01 in all cases. (Online version in colour.)

Figures 8 and 9 illustrate the far-field stress directivity patterns associated with the scattered fields from the co-void system. We focus on two measures of this; firstly $\sigma_{D}/(\lambda +2\mu )$, where $\sigma_{D}=\text{tr}(\sigma )/3$ is the dilatational stress, and $\sigma_{r\theta }/(\lambda +2\mu )$, where $\sigma_{r\theta }$ is the shear stress. It is straightforward to show that the far-field directivity pattern of $\sigma_{D}/(\lambda +2\mu )$ is given by (1−4ξ/3)g(θ) where g(θ) is the far-field directivity pattern of $\varphi^{\text{sc}}$ as defined by (B 10). The far-field directivity associated with the scaled shear stress is simply ξ times that of $\psi^{\text{sc}}$, where the directivity pattern of the latter is defined in similar fashion to $\varphi^{\text{sc}}$ .

Figure 8 illustrates these two measures of directivity for the case of touching voids at their resonant frequency of $k_{s}a=0.5\;(\lambda_{p}/a=125.7)$; we find that the directivity patterns are largely independent of the angle of incidence so that only normal incidence is illustrated. Figure 9 illustrates the directivity patterns for $k_{s}a=0.86(\lambda_{p}/a=73.1),d/a=47.3$, which represents the configuration at which the cross-section of the co-void system is a maximum at normal incidence. Four values of incidence are chosen $\theta_{p}=0,30,60$ and $90^{\circ }$. The directivity patterns are now strongly dependent upon the angle of incidence and in this case, shear-stress directivity is much less significant than in the case of touching voids illustrated in figure 8.

### (c) Monopole field approximations

Ivansson [24] found that for frequencies close to the GMR, the multiple scattering problem for a low stiffness polymer is dominated by monopole terms of the individual scatterers. Hence here, in addition to determining the SCS via the expression in (B 18) with J=2, which includes all coupling due to multiple scattering, we also consider two monopole approximations. The first, which we shall term the *simple* monopole approximation (SMA) and the second which we shall term the *full* monopole approximation (FMA). In the FMA, the full solution of the multiple scattering problem is evaluated but only the monopole terms are retained in the expression (B 18) for the SCS.

In the SMA, we note that if monopole terms only are significant in the multiple scattering problem then for an incident compression wave, shear waves become negligible since compression and shear waves are decoupled at angular order zero, that is $T_{12}^{\left(0\right)}=T_{21}^{\left(0\right)}=0$. In this approximation, the only non-zero scattering coefficients for the co-void problem are $B_{0}^{\left(1\right)}$ and $B_{0}^{\left(2\right)}$, which are obtained easily from (2.32) and (2.5) with $R_{1}^{\left(2\right)}=R_{2}^{\left(1\right)}=d$; they are found to be

3.1 $$B_{0}^{\left(1\right)}\approx q_{p}T_{11}^{\left(0\right)}\left(\frac{e^{-\text{i}\alpha_{p}}+e^{\text{i}\alpha_{p}}G}{1-G^{2}}\right)$$

and

3.2 $$B_{0}^{\left(2\right)}\approx q_{p}T_{11}^{\left(0\right)}\left(\frac{e^{\text{i}\alpha_{p}}+e^{-\text{i}\alpha_{p}}G}{1-G^{2}}\right),$$

where

3.3 $$\alpha_{p}=\frac{k_{p}d}{2}\sin\theta_{p}\;\text{and}\;G=T_{11}^{\left(0\right)}H_{0}^{\left(1\right)}(k_{p}d).$$

Inserting (3.1) and (3.2) into (B 18) (retaining just the monopole terms), we find, noting $\phi_{1}=-\pi /2$, $\phi_{2}=\pi /2$ and $d_{1}=d_{2}=d/2$, that

3.4 $$\Gamma_{2}^{\left(0\right)}=-\frac{8}{k_{p}}ℜ\left\{T_{11}^{\left(0\right)}\left(\frac{1+\cos(k_{p}d\sin\theta_{p})G}{1-G^{2}}\right)\right\},$$

where the superscript (0) indicates that only the angular order zero (monopole) term is used.

In §2b, we saw that $|T_{11}^{\left(0\right)}|$ has a maximum value of unity. Thus, for large $k_{p}d$, which would be the case for well separated voids, G→0, and

3.5 $$\Gamma_{2}^{\left(0\right)}\approx -\frac{8}{k_{p}}ℜ\{T_{11}^{\left(0\right)}\},\;k_{p}d\to \infty ,$$

which is just twice the SCS of (2.14) for a single, isolated void under the SMA, as should be expected.

Consider now, the co-void cross-section at the GMR frequency of a single void, corresponding to $k_{p}=k_{0}$ say, at which value $T_{11}^{\left(0\right)}=-1$ for ξ≤0.075. Denoting the co-void SCS at the single void GMR by $\Gamma_{2}(GMR)$ we find from (3.4) that for the SMA,

3.6 $$\Gamma_{2}^{\left(0\right)}(\text{GMR})=\frac{8}{k_{0}}ℜ\left\{\frac{1-\cos(k_{0}d\sin\theta_{p})H_{0}^{\left(1\right)}(k_{0}d)}{1-{\left(H_{0}^{\left(1\right)}(k_{0}d)\right)}^{2}}\right\}.$$

Recalling that for a single void, $\Gamma_{1}^{\left(0\right)}(\text{GMR})=4/k_{0}$, we observe that for a given angle of incidence, $\theta_{p}$, the ratio $\Gamma_{2}^{\left(0\right)}(\text{GMR})/\Gamma_{1}^{\left(0\right)}(\text{GMR})$ depends only upon $k_{0}d$.

For $k_{0}d\ll 1$, (3.6) becomes independent of $\theta_{p}$; at frequencies close to the GMR this would occur if the two voids were closely spaced within an elastic material of low shear modulus. For such a material, $\xi =\mu /(\lambda +2\mu )=k_{p}^{2}/k_{s}^{2}\ll 1$, and at frequencies near the GMR frequency, $k_{s}a=0(1)$. Hence at the GMR, $k_{0}d=k_{p}d=0(\sqrt{\xi }\;d/a)$, noting that d/a≥2. In this limit, $J_{0}(k_{0}d)∼1$ and $Y_{0}(k_{0}d)\gg 1$, whence (3.6) acquires the approximate form,

3.7 $$\Gamma_{2}^{\left(0\right)}(\text{GMR})\approx \Gamma_{1}^{\left(0\right)}(\text{GMR})\left[\frac{4}{4+Y_{0}^{2}(k_{0}d)}\right],\;k_{0}d\ll 1,\;\xi ≲0.075,$$

and we see that for $k_{0}d\ll 1,\;\Gamma_{2}^{\left(0\right)}(GMR)$ could be substantially smaller than $\Gamma_{1}^{\left(0\right)}(GMR)$.

**Figure 10.**
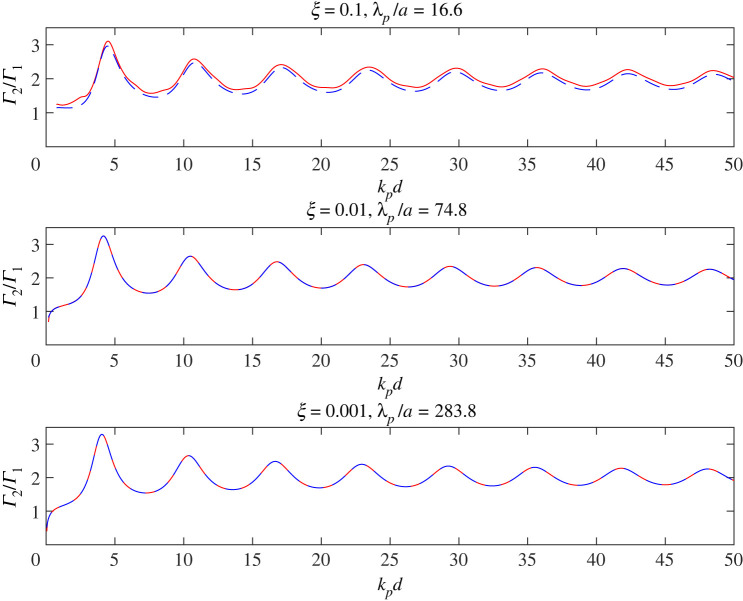
Non-dimensionalized SCS versus $k_{p}d$ for two voids insonified normal to their separation by a plane compression wave at the GMR frequency of a single void. The value of rigidity and the associated value of $\lambda_{p}/a$ at the GMR frequency is indicated on each sub-plot. For each value of rigidity, the co-void cross-section is normalized by that of a single void at the same frequency. Red solid-lines are calculated using the full expression for the SCS, (B 18), while blue dashed-lines show the SMA of (3.4). (Online version in colour.)

Figure 10 shows the co-void SCS for normally incident compression waves at the GMR frequency of a single void, plotted as a function of $k_{p}d$ for three different values of the rigidity parameter, ξ. For each value of ξ, the GMR frequency is taken to be the value at the peak of the single void cross-section shown in figure 2*d*, where the corresponding value of $\lambda_{p}/a$ is indicated. The co-void cross-section is calculated in two ways: from the ‘full’ expression of (B 18) with J=2 (red solid-lines), and also using the SMA of (3.4) (blue dashed-lines). The plotted values are scaled on the corresponding single void cross-sections of figure 2*d*. All three plots show very similar behaviour. Moreover, the monopole approximation is very close to the full form especially for ξ=0.01 and ξ=0.001; ξ=0.1 is perhaps close to the upper limit of validity of the monopole approximation.

**Figure 11.**
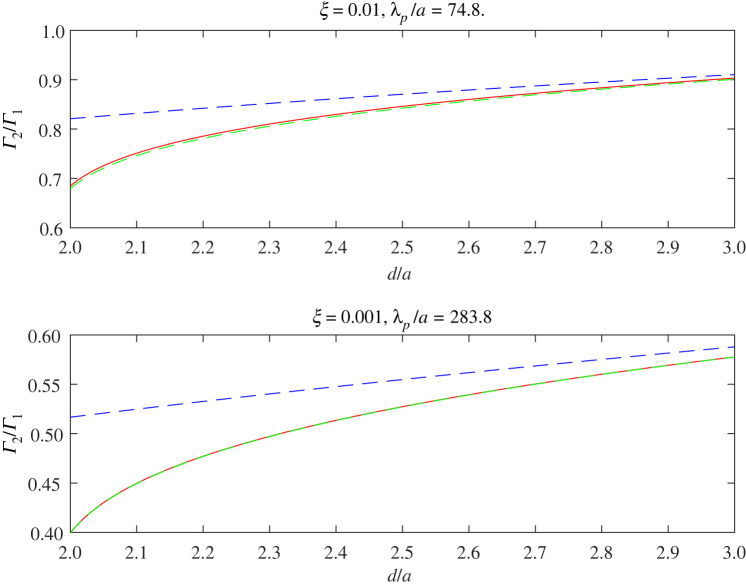
Non-dimensionalized SCS versus d/a for two voids insonified normal to their separation by a plane compression wave at the GMR frequency of a single void. The value of rigidity and the associated value of $\lambda_{p}/a$ at the GMR frequency is indicated on each sub-plot. For each value of rigidity, the co-void cross-section is normalized by that of a single void at the same frequency. Red solid-lines are calculated using the full expression for the SCS, (B 18), while blue dashed-lines show the SMA of (3.4). Also, shown in green, is the contribution of the monopole term alone from the full solution. (Online version in colour.)

For all three values of ξ, the co-void cross-section peaks at $k_{p}d∼4$. At increasingly larger values of $k_{p}d$, $\Gamma_{2}/\Gamma_{1}$ oscillates with decreasing amplitude about a value of 2, tending towards the latter as $k_{p}d$ becomes large—much as expected from previous discussions. Conversely, as $k_{p}d$ reduces in value, the co-void cross-section falls; indeed for ξ=0.01 and ξ=0.001, we see that at very small values of $k_{p}d$, $\Gamma_{2}(\text{GMR})$ becomes less than $\Gamma_{1}(\text{GMR})$, as suggested by (3.7). This behaviour is illustrated more clearly in figure 11, which illustrates the co-void cross-section at the GMR frequency as a function of d/a≥2 for a normally incident plane compressional wave. At very close separation, the SMA of (3.4) becomes inaccurate and overestimates the true value indicated by the full expression of (B 18). The green-dashed curve shows the corresponding SCS from the FMA, i.e. the contribution to the SCS of the monopole term, where the latter is obtained by solving the full multiple scattering problem with angular orders lying between ±8 inclusive, for both compressional and shear waves. Even when the voids are touching, the SCS is still dominated by the monopole term of the full solution but the SMA is inaccurate for d/a≲3.

### (d) Equivalent void for a co-void metacluster

As described in §3a, one can compare the resonance achieved by a metacluster with that arising from a single void. We could also try to assign an ER to the metacluster. Clearly, such a resonator cannot exactly match the scattered response of a metacluster in terms of its resonant response, its far-field scattering pattern and SCS, but some properties could be retained in order to approximate scattering in multiple scattering formulations for example. We stress here then that in terms of equivalence we mean therefore only equivalance in terms of its low frequency response. As we shall see, at other frequencies other non-physical effects can arise. Here, we consider three approximations:

— the SMA;
— an equivalent void, with the same volume;
— an equivalent resonance, i.e. a circular void with a volume chosen to match the resonance.

Above we noted that the co-void metacluster achieved a lower resonant frequency than a single void of the same volume, or rather, if the co-void configuration is configured to achieve the same resonant frequency as a particular single void then it does so with a smaller overall volume. The first two approximations above are straightforward to plot given the discussion above. For the third approximation, we must determine the radius of the equivalent single void matching the resonant frequency of the touching co-void system. For ξ=0.01, this is $\tilde{a}\approx (0.84/0.5)a\approx 1.68a$. Hence the resonant frequency of a single void can be matched by two touching voids but with a volume reduction of almost 30%.

**Figure 12.**
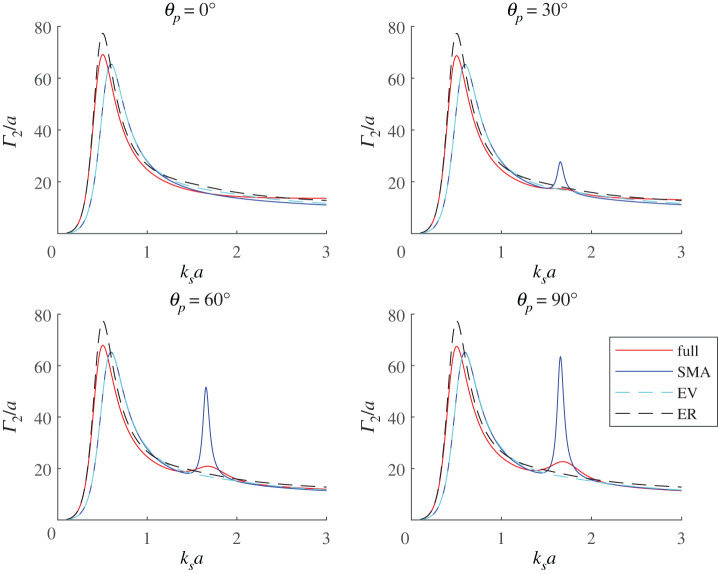
Non-dimensionalized SCS, $\Gamma_{2}/a$, versus $k_{s}a$ for two touching voids in a substrate of rigidity ξ=0.01. The voids are insonified by a plane wave at various angles of incidence, $\theta_{p}$, as indicated on each sub-plot. Red solid-lines are calculated using the full expression for the SCS, (B 18), while blue solid-lines show the SMA of (3.4). The curves denoted ‘EV’ show the cross-section of a single void have the same volume as the two voids combined, that is a single void of radius $\sqrt{2}a$; the curves denoted ‘ER’ show the cross-section for a singe void whose GMR frequency matches that of the two touching voids, that is a void with a radius of ≈1.68a. (Online version in colour.)

In figure 12, we plot the various approximations to the touching co-void resonator and indicate the effect at four angles of incidence. The dependence on angle is important because clearly any such single *equivalent* void will not possess such a dependence. It is notable that the SMA and the equivalent volume (EV) void are almost identical in the regime $k_{s}a≲1$ for all angles of incidence. At larger values however the SMA accentuates the milder secondary resonance in the full system as $\theta_{p}$ increases from 0 to $90^{\circ }$. The equivalent resonator (ER), with larger radius than EV, matches the resonant frequency of the co-void (by design) but has a larger resonant amplitude.

## 4. Metaclusters with more complex configurations

The co-void resonator illustrates that local, near-field shear effects can be exploited to modify the resonance even in the long compressional wavelength regime. We now briefly illustrate some results for more complex metaclusters. The results indicate that there is rich behaviour, which could be studied in much greater depth in order to design elastodynamic resonators with tailored resonances and scattering responses.

### (a) Tri-void metacluster

**Figure 13.**
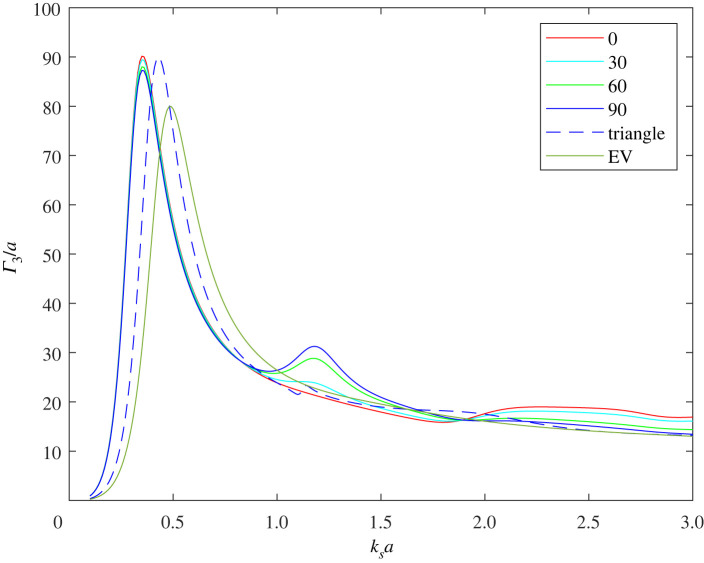
$\Gamma_{3}/a$ for various tri-void configurations: in-line at $0\to 90^{\circ }$ angles of incidence; a triangular configuration (largely independent of the incidence angle). Also shown is a single void of equivalent volume, i.e. a radius of $\sqrt{3}a$ labelled ‘EV’. (Online version in colour.)

Consider, first, three voids of equal radius and let us illustrate how their respective proximity affects the resulting GMR. In figure 13, we plot the normalized SCS, $\Gamma_{3}/a$, for three touching voids in a line, when insonified by a compression wave at various angles of incidence—as before $0^{\circ }$ is normal to the line of voids. The resonant frequency is identical for all angles of incidence, although the magnitude of $\Gamma_{3}$ varies slightly. Also shown in figure 13 is the SCS when the three voids are arranged in a triangular configuration (touching)—with centres at $\left(x,y)=(-a,-a/\sqrt{3}),(a,-a/\sqrt{3}),(0,2a/\sqrt{3}\right)$. In this case, the magnitude of the SCS varies very little with the angle of incidence and therefore the figure shows only the $0^{\circ }$ incidence case. The resonant frequency of the triangle configuration is slightly higher than in the case of the line array of three voids, but both are lower than the resonant frequency of a single void of the same volume—denoted ‘EV’ in the figure and having a radius of $\sqrt{3}a$.

**Figure 14.**
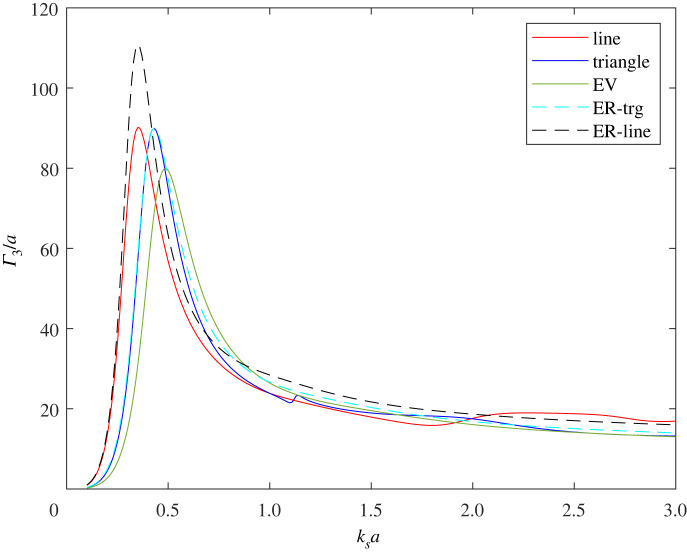
$\Gamma_{3}/a$ at $0^{\circ }$ angle of incidence for three touching voids in a line and in a triangular configuration. Also shown is the cross-section for a single void of the same volume (EV), a single void having the same resonant frequency as the in-line configuration (ER-line), and a single void having the same resonant frequency as the triangle configuration (ER-trg). (Online version in colour.)

A single void of radius ≈2.4a would achieve the same resonant frequency as the three voids in-line, therefore the latter achieves the same resonant frequency as a single void but with a volume reduction of about 48%. For the triangle configuration, the volume reduction to achieve a given resonant frequency value is about 20% compared to a single void. This is illustrated in figure 14, which shows the SCS at $0^{\circ }$ angle of incidence for: three voids in-line, the triangle configuration, a single void of the same volume (EV), a single void having the same resonant frequency as the in-line configuration (ER-line), and a single void having the same resonant frequency as the triangle configuration (ER-trg). It is interesting to note that the behaviour of the latter is almost identical to that of the triangle configuration despite the difference in volume.

### (b) Quad-void metacluster

**Figure 15.**
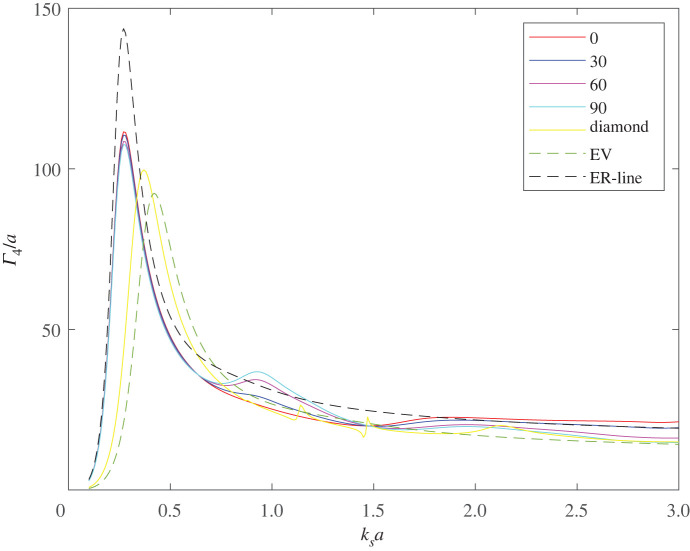
The non-dimensionalized SCS, $\Gamma_{4}/a$, for four touching voids arranged in-line at incidence angles ranging from 0 to $90^{\circ }$. The SCS is also shown at $0^{\circ }$ for: four voids arranged in a diamond configuration (touching); a single void of the same volume (EV); and a single void having the same resonant frequency as the in-line configuration (ER-line), that is a void with radius 3.111a. (Online version in colour.)

The final configuration illustrated here is the case of *four* voids. Figure 15 shows the non-dimensionalized SCS, $\Gamma_{4}/a$, for four touching voids of equal radius, arranged in an in-line along the y-axis. The cross-section is illustrated for various angles of incidence ranging from 0 to $90^{\circ }$, whereas above, the $0^{\circ }$ case is normal to the line of voids and $90^{\circ }$ is at grazing incidence to the array. As with the co-void and tri-void configurations, the resonant frequency is the same for all incidence angles but there is some small variation in the magnitude of the SCS; any variation in magnitude is expected to be small due the compactness of the configuration with respect to the compressional wave. The SCS is also shown for: four touching voids arranged in a diamond configuration with centres at $\left(x,y)=(-a,0),(a,0),(0,\sqrt{3}a),(0,-\sqrt{3}a\right)$; a single void of the same volume (EV), that is with a radius of 2a; and a single void having the same resonant frequency as the in-line configuration (ER-line), that is a void with radius ≈3.111a. The diamond configuration shows very little variation with angle of incidence and only the $0^{\circ }$ incidence case is shown.

As we found in the tri-void example, the in-line configuration has a substantially lower resonant frequency than the more compact diamond geometry and both are lower than a single void of the same volume. A single void having the same resonant frequency as the in-line configuration gives a substantially higher SCS but the in-line configuration achieves the same resonant frequency with a volume reduction of about 59%.

**Figure 16.**
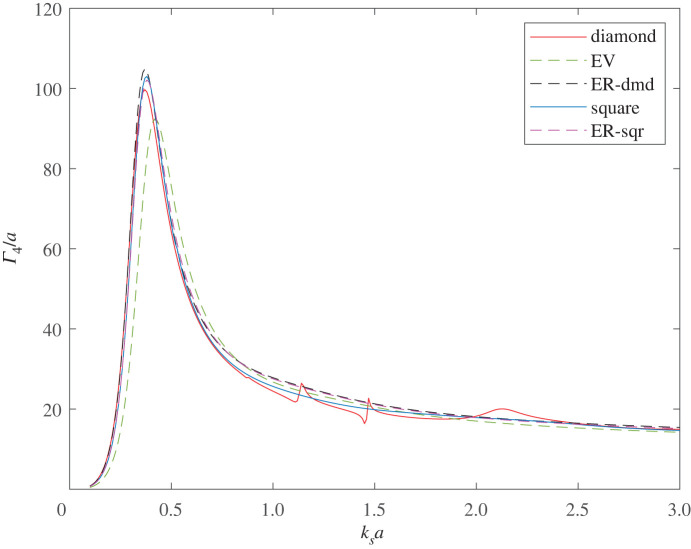
The non-dimensionalized SCS, $\Gamma_{4}/a$, at $0^{\circ }$ incidence for four touching voids arranged in diamond and square configurations. Also shown is the SCS of a single void having: the same volume as the four voids (EV), the same resonant frequency as the diamond configuration (ER-dmd) and the same resonant frequency as the square configuration (ER-sqr). (Online version in colour.)

Figure 16 compares the SCS of the diamond configuration with that of four voids arranged in a square with centres at (x,y)=(−a,−a),(a,−a),(−a,a),(a,a); the cross-sections are shown for $0^{\circ }$ compressional wave incidence. There is little difference between the two configurations although the resonant frequency of the diamond configuration is slightly lower than that of the square and the value of $\Gamma_{4}$ is slightly lower for the diamond. Also shown are the SCSs of single voids having the same resonant frequency as the diamond (ER-dmd) and square (ER-sqr), and the EV void is shown again for comparison. For a given resonant frequency, the diamond configuration gives a volume reduction of about 22% compared with a single void, while the square configuration achieves a volume reduction of about 18%. Interestingly, the square configuration achieves this with a fractionally higher SCS value, while in the case of the diamond, the SCS is about 5% lower than that of the single void.

## 5. Conclusions

Resonance resides at the heart of metamaterial science. Elastodynamic control using elastic resonators is a topic that has received very little attention compared to its electromagnetic and acoustic counterparts. The study here then is important given that it begins to explore the mechanisms by which the GMR, a well-known effect associated with a single void in a low shear material, can be manipulated by placing multiple voids in close proximity and exploiting local shear effects.

We illustrated that at frequencies in the vicinity of the GMR of a single void in a substrate of low rigidity—ξ≲0.1—the multiple scattering problem for N identical voids, insonified by a plane compression wave, is dominated by the monopole term of the compression-wave scattered by individual voids. This dominance is such that provided the voids are not too close, only compressional wave monopole terms need be considered within the multiple scattering solver, i.e. the scattering problem is identical to that of a fluid. Despite this fact, when the voids are close, i.e. d/a≲3, higher order modes for both compressional and shear waves become significant in terms of multiple scattering and must be incorporated for accurate solutions. Even in this latter case, however, the SCS for an incident compressional wave is dominated by the compressional wave monopole terms of the individual voids, but the higher order compressional and shear wave modes mentioned above are necessary to obtain the correct value for the monopole scattering coefficients due to near-field coupling.

The resonant frequency of a cluster of touching voids, defined as the peak of the SCS, is generally lower than that of single void, substantially so if the voids are configured in a line. The in-line void configurations show more dependence upon angle of incidence than clusters with more symmetric arrangements—e.g. triangle, diamond, square etc. The combination of such metaclusters is therefore potentially of great interest with regard to how the N-void metaclusters can be distributed to create directional, resonant elastodynamic metamaterials.

A consequence of the results obtained herein is that a desired resonant frequency value can be obtained with a substantial volume reduction compared with a single void by combining several voids into a cluster. For example, the resonant frequency of a single void can be matched by two touching voids but with a volume reduction of almost 30%. The penalty for this is a lower value of the SCS compared with a single void.

We illustrated the concept for some simple configurations and this resonance could be exploited in many different ways using combinations of void sizes, enabling for example a broader, lower frequency response.

The far-field directivity patterns of the cluster are dependent upon the number and configuration of voids; if the configuration is compact with respect to the compression wave then the far-field directivity of the latter (and associated quantities such as radial displacement and dilatational stress) is relatively omni-directional; this is a consequence of the individual void scattering coefficients being monopole dominated. The far-field directivity patterns of quantities associated with shear (shear potential function, polar displacement, shear-stress) are more directional because of the much shorter wavelength of these waves but the far-field shear wave amplitudes are much lower in magnitude than those associated with the compressional wave because of the low rigidity value of the substrate.

Future work points to alternative resonant mechanisms in elasticity and designing metaclusters for a specific macroscopic, effective elastic metamaterial response across desired frequency ranges.

## Data Availability

This article has no additional data.

## Appendix A. Scattering coefficients for an isolated circular void

Scattering from a circular void is subject to the traction-free boundary condition encompassed by (2.6) to (2.8). By writing the potential functions as the sum of incident and scattered fields as expressed by (2.3) and (2.4), we find after some straightforward if tedious manipulation that the coefficients of the scattering matrix, defined by (2.5), are given as follows denoting $\kappa =k_{p}a$ and $\chi =k_{s}a$,

A 1 $$\begin{array}{rl} T_{11}^{\left(m\right)} & \;=\frac{\left\{m^{2}A^{2}M^{2}\kappa \chi J_{m}^{\prime }(\kappa )H_{m}^{{\left(1\right)}^{\prime }}(\chi )-[J_{m}(\kappa )+M\kappa J_{m}^{\prime }(\kappa )][H_{m}^{\left(1\right)}(\chi )+M\chi H_{m}^{{\left(1\right)}^{\prime }}(\chi )]\right\}}{D_{m}}, \end{array}$$

A 2 $$\begin{array}{rl} T_{12}^{\left(m\right)} & \;=-\frac{2\xi mAM}{\pi D_{m}},\;T_{21}^{\left(m\right)}=\frac{2\;mAM}{\xi \pi D_{m}} \end{array}$$

A 3 $$\begin{array}{rl} \mathrm{and}\;\;T_{22}^{\left(m\right)} & \;=\frac{\left\{m^{2}A^{2}M^{2}\kappa \chi J_{m}^{\prime }(\chi )H_{m}^{{\left(1\right)}^{\prime }}(\kappa )-[J_{m}(\chi )+M\chi J_{m}^{\prime }(\chi )][H_{m}^{\left(1\right)}(\kappa )+M\kappa H_{m}^{{\left(1\right)}^{\prime }}(\kappa )]\right\}}{D_{m}}, \end{array}$$

where

A 4 $$\begin{array}{rl} D_{m} & \;=[H_{m}^{\left(1\right)}(\kappa )+M\kappa H_{m}^{{\left(1\right)}^{\prime }}(\kappa )][H_{m}^{\left(1\right)}(\chi )+M\chi H_{m}^{{\left(1\right)}^{\prime }}(\chi )]-m^{2}A^{2}M^{2}\kappa \chi H_{m}^{(1){\;}^{\prime }}(\kappa )H_{m}^{{\left(1\right)}^{\prime }}(\chi ), \end{array}$$

where

A 5 $$A=\left[1+\frac{2(1-m^{2})}{\chi^{2}}\right]\;\mathrm{and}\;M=\frac{2\chi^{2}}{\left[\chi^{2}-2m(m+1)][\chi^{2}-2m(m-1)\right]}.$$

Primes on Bessel functions denote their derivatives, for example $J_{m}^{\prime }(z)=dJ_{m}(z)/dz$. Finally, the following symmetry properties are noted:

A 6 $$T_{11}^{\left(-m\right)}=T_{11}^{\left(m\right)},\;T_{12}^{\left(-m\right)}=-T_{12}^{\left(m\right)},\;T_{21}^{\left(-m\right)}=-T_{21}^{\left(m\right)}\;\mathrm{and}\;T_{22}^{\left(-m\right)}=T_{22}^{\left(m\right)}.$$

## Appendix B. Two-dimensional elastic scattering cross-sections

The SCS for a two-dimensional object^1^ is actually a length, although the term cross-section appears to be commonly used within the literature. It is defined with respect to an incident plane wave as the average power per unit length (out of plane) scattered by the two-dimensional object divided by the intensity of the incoming wave.

For harmonic compressional/shear excitation in the *x*–*y* plane, Barratt & Collins [29] show that the average rate at which energy is transmitted across unit length (in the z-direction) of a cylindrical surface of circumference C in the x–y plane is given by

B 1 $$R=-\frac{\omega }{2}ℜ\left\{\text{i}\int_{C}(\sigma_{rr}{\bar{u}}_{r}+\sigma_{r\theta }{\bar{u}}_{\theta })\text{d}l\right\},$$

where over-bars denote complex conjugation, and $\sigma_{rr}$, $\sigma_{r\theta }$, $u_{r}$ and $u_{\theta }$ are some general polar stresses and displacements defined in terms of scalar potentials via the expressions (2.7) to (2.9). Taking C to be a cylindrical contour of radius r, dl=r dθ this takes the form

B 2 $$R=\frac{\omega r}{2}\;ℑ\left\{\int_{0}^{2\pi }(\sigma_{rr}{\bar{u}}_{r}+\sigma_{r\theta }{\bar{u}}_{\theta })\text{d}\theta \right\}.$$

Consider now a single, circular scatterer contained entirely within C. Without loss of generality, we can assume that the origin of the scatterer is coincident with that of C, whence for the scattered field the potential functions are given by (2.4). Inserting these expressions into (2.7) to (2.9) and then into (B 2), yields the following expression for the energy scattered by an inclusion that is embedded within an undamped elastic material, noting that $k_{p}$, $k_{s}$ and ξ are real,

B 3 $$R_{\text{sc}}=(\lambda +2\mu )\frac{2\omega }{k_{p}^{2}}\sum_{m=-\infty }^{\infty }[\xi^{2}|D_{m}{|}^{2}+|B_{m}{|}^{2}].$$

We take the incident plane wave to be the compression wave defined by (2.10). Its intensity, that is the average rate at which energy is transmitted across unit area perpendicular to its direction of propagation, is independent of $\theta_{p}$, which may therefore be set to zero. In which case the intensity can be written

B 4 $$I=\frac{\omega }{2}ℑ\{\sigma_{xx}{\bar{u}}_{x}\},$$

where

B 5 $$\sigma_{xx}=(\lambda +2\mu )\varphi^{\text{in}}\;\mathrm{and}\;u_{x}=\frac{-\text{i}}{k_{p}}\varphi^{\text{in}}.$$

Therefore,

B 6 $$I=(\lambda +2\mu )\frac{\omega |A{|}^{2}}{2k_{p}},$$

and the elastic SCS for the incident compression wave is given by

B 7 $$\Gamma =\frac{R_{\text{sc}}}{I}=\frac{4}{k_{p}}\sum_{m=-\infty }^{\infty }[\xi^{2}{\left|\frac{D_{m}}{A}\right|}^{2}+{\left|\frac{B_{m}}{A}\right|}^{2}],$$

noting that $|B_{m}/A|$ and $|D_{m}/A|$ are the magnitudes of the Fourier decomposed, elastic-scattering coefficients for a unit amplitude, incident, plane compression-wave.

While the form (B 7) is readily evaluated for single scatterers, an alternative form that is more easily applied to scattering by multiple objects was derived by Barratt & Collins [29] who note that for an non-absorbing object within an undamped elastic material the total energy *R* defined in (B1) is zero, and therefore the scattered energy flux per unit length can be written as the interaction between the incident and scattered fields (see their equation (3.8)), *viz*

B 8 $$R_{\text{sc}}=-\frac{\omega }{2}\int_{C}ℑ\{\sigma_{rr}^{\text{in}}{\bar{u}}_{r}^{\text{sc}}+\sigma_{r\theta }^{\text{in}}{\bar{u}}_{\theta }^{\text{sc}}+\sigma_{rr}^{\text{sc}}{\bar{u}}_{r}^{\text{in}}+\sigma_{r\theta }^{\text{sc}}{\bar{u}}_{\theta }^{\text{in}}\}\;\text{d}l,$$

where the superscript ‘sc’ denotes quantities associated with the scattered field, while ‘in’ denotes quantities associated with the incident field. (B 8) is analogous to what Martin [33] refers to as the extinction cross-section in acoustics noting that for a non-absorbing scatterer the extinction and elastic cross-sections are equal.

Using (B 8) Barratt and Collins go on to show that the SCS is related to the far-field scattering amplitude, g(θ), for a unit-amplitude, incident, compressional wave *viz*,

B 9 $$\Gamma =-\frac{4}{k_{p}}ℜ\{g(\theta_{p})\}.$$

g(θ) is defined by the following expression,^2^

B 10 $$\varphi^{\text{sc}}(r,\theta )∼g(\theta )H(k_{p}r),\;r\to \infty ,$$

where

B 11 $$H(z)=\sqrt{\frac{2}{\pi z}}\;e^{\text{i}(z-(\pi /4))}.$$

It is clear from (2.28) that for J voids contained within a bounded region

B 12 $$\varphi^{\text{sc}}(r,\theta )=\sum_{j=1}^{J}\sum_{m=-\infty }^{\infty }\left(\frac{B_{m}^{\left(j\right)}}{A}\right)H_{m}^{\left(1\right)}(k_{p}r_{j})\;e^{\text{i}m\theta_{j}},$$

where to maintain consistency with notation used previously, $B_{m}^{\left(j\right)}$ denotes the Fourier decomposed scattering amplitudes of void j when insonification is by a plane compression wave of amplitude A, whereas (B 9) and (B 10) require A=1, and we recall that $\left(r_{j},\theta_{j}\right)$ are the polar coordinates of the field point expressed in the jth local coordinate system.

The jth local coordinates are related to global coordinates (r,θ) through

B 13 $$r_{j}=\sqrt{r^{2}+d_{j}^{2}-2rd_{j}\cos(\phi_{j}-\theta )}\;\mathrm{and}\;\theta_{j}=\arctan\left(\frac{r\sin\theta -d_{j}\sin\phi_{j}}{r\cos\theta -d_{j}\cos\phi_{j}}\right),$$

where $\left(d_{j},\phi_{j}\right)$ are the polar coordinates of the centre of the jth void within the global coordinate system. For $r\gg d_{j}$,

B 14 $$r_{j}=r-d_{j}(\cos(\phi_{j}-\theta )+O(d_{j}/r))$$

and

B 15 $$\theta_{j}=\theta +O(d_{j}/r).$$

As r and hence $r_{j}\to \infty$ we may use the large argument form for the Hankel function, $H_{m}^{\left(1\right)}(z)∼H(z)\;e^{-\text{i}m\pi /2}$ [31], in (B 12) provided the angular order summation is truncated at some suitable point ±M say. In which case, by inserting (B 14) and (B 15) into (B 12), we find

B 16 $$\varphi^{\text{sc}}(r,\theta )∼H(k_{p}r)\sum_{j=1}^{J}\sum_{m=-M}^{M}\left(\frac{B_{m}^{\left(j\right)}}{A}\right){\left(-\text{i}\right)}^{m}\;e^{\text{i}m\theta }\;e^{-\text{i}k_{p}d_{j}\cos(\phi_{j}-\theta )},\;r\to \infty .$$

Comparing the above with (B 10), we see that

B 17 $$g(\theta )=\sum_{j=1}^{J}\sum_{m=-M}^{M}\left(\frac{B_{m}^{\left(j\right)}}{A}\right){\left(-\text{i}\right)}^{m}\;e^{\text{i}m\theta }\;e^{-\text{i}k_{p}d_{j}\cos(\phi_{j}-\theta )},$$

and hence, the SCS for J scatterers is given by

B 18 $$\Gamma_{J}=-\frac{4}{k_{p}}\sum_{j=1}^{J}\sum_{m=-M}^{M}ℜ\left\{\frac{B_{m}^{\left(j\right)}}{A_{m}^{\left(j\right)}}\right\},$$

where

B 19 $$A_{m}^{\left(j\right)}=A\;e^{\text{i}k_{p}d_{j}\cos(\phi_{j}-\theta_{p})}{\text{i}}^{m}\;e^{-\text{i}m\theta_{p}}.$$

We note that an incident plane compression wave, propagating at angle $\theta_{p}$ to the x-axis, has the form

B 20 $$\varphi^{\text{in}}(r,\theta )=A\;e^{\text{i}k_{p}r\cos(\theta -\theta_{p})},$$

and hence, $A_{m}^{\left(j\right)}$ are the Fourier expansion coefficients of the incident field about the centre coordinates of the jth void, and $B_{m}^{\left(j\right)}/A_{m}^{\left(j\right)}$ are the effective scattering coefficients for the jth scatterer in the presence of multiple voids.
